# Rupture de l’albuginée du corps caverneux

**DOI:** 10.11604/pamj.2017.27.210.13104

**Published:** 2017-07-20

**Authors:** Hamza Dergamoun, Zayd El Boukili El Makhoukhi

**Affiliations:** 1 Université Mohammed 5, Faculté de Médecine et de Pharmacie de Rabat, Hopital Ibn Sina, Service d’Urologie A, Maroc

**Keywords:** Fracture de la verge, hématome, albuginée, Fracture of the penis, haematoma, albuginea

## Abstract

The fractures of the penis are rare in our context and are most often secondary to false penile fracture. A more common cause has been described in the countries of the Middle East where people interrupt the erection due to a violent bending of their penis called “Taqaandan”. We here report the case of a 21-year old patient with no particular previous history, presenting to the emergency department at the Medical Center Avicenna with pain and deformation of the penis following “Taqaandan” manoeuvre. After having heard a click the patient felt pain and had violent detumescence without associated urinary problems, thus excluding urethral involvement. The image shows the classic “eggplant” appearance (A). Ultrasound confirmed the diagnosis of rupture of the albuginea of the corpus cavernosum, and the patient underwent surgery. A selective contralateral approach was used in order to avoid unnecessary dissection. After the evacuation of the haematoma, the fracture line was shown (B) and then fixed with reverse suture. The patient was discharged the day after surgery and was put under analgesic and antihormonal treatment. The patient went for a check-up after 2, 4 and 6 months and showed recovery of normal erectile function without cranking or fibrous plate.

## Image en médecine

Les fractures de la verge sont peu fréquentes dans notre contexte, et font le plus souvent suite à un faux pas du coït. Une cause plus fréquente a été décrite dans les pays du moyen orient qui vise à interrompre l'érection par une flexion brutale de la verge, appelée « Taqaandan ». Nous rapportons ici le cas d'un patient de 21 ans, sans antécédent particulier, qui se présente aux urgences du centre hospitalier Avicenne pour une douleur et déformation de la verge suite à une manœuvre de « Taqaandan ». Après avoir entendu un claquement, le patient a noté une douleur et une détumescence brutale, sans signes urinaires associés excluant alors une atteinte urétrale. Nous remarquons sur l'image l'aspect classique en aubergine (A). Après réalisation d'une échographie confortant de diagnostic de rupture de l'albuginée du corps caverneux, le patient a été admis au bloc opératoire ou il a bénéficié d'une intervention chirurgicale. Nous avons alors choisis un abord sélectif controlatéral à la déviation, afin d'éviter toute dissection inutile. Après évacuation de l'hématome, le trait de fracture a été visualisé (B) puis suturé par des points inversés. Le patient a été déclaré sortant le lendemain de l'intervention sous traitement antalgique et traitement anti hormonal. Notre jeune patient a été revu à 2, 4 et 6 mois en ayant récupéré une fonction érectile normale sans coudure ni plaque de fibrose.

**Figure 1:**
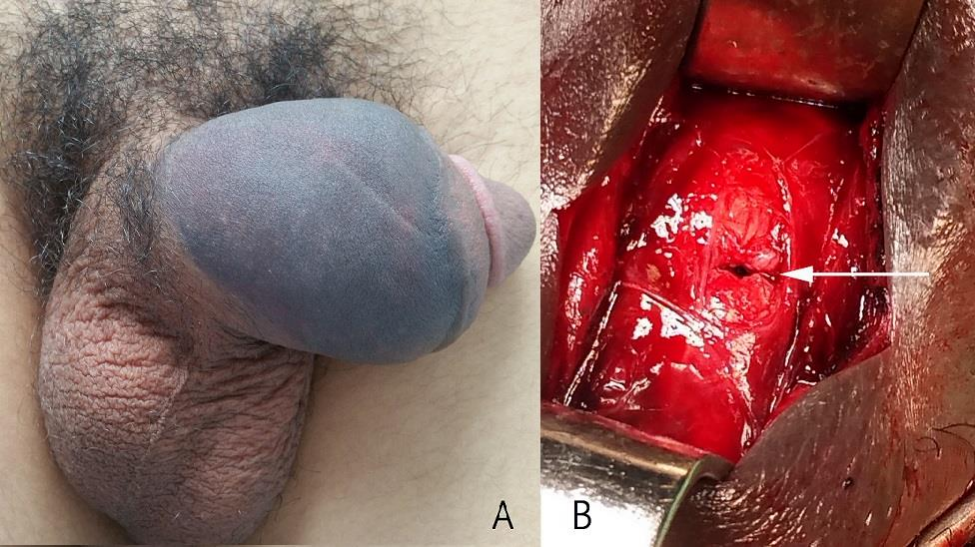
A) aspect classique en aubergine, avec repérage de la rupture du corps caverneux; B) après évacuation de l’hématome

